# Effects of Interfaces on the Structure and Novel Physical Properties in Epitaxial Multiferroic BiFeO_3_ Ultrathin Films

**DOI:** 10.3390/ma7075403

**Published:** 2014-07-23

**Authors:** Chuanwei Huang, Lang Chen

**Affiliations:** Department of Physics, South University of Science and Technology of China, Shenzhen 518055, China; E-Mail: huang.cw@sustc.edu.cn

**Keywords:** BiFeO_3_ ultrathin film, misfit strain, depolarization field, phase transition, domain pattern, strain-induced morphotropic phase boundary (MPB)

## Abstract

In functional oxide films, different electrical/mechanical boundaries near film surfaces induce rich phase diagrams and exotic phenomena. In this paper, we review some key points which underpin structure, phase transition and related properties in BiFeO_3_ ultrathin films. Compared with the bulk counterparts, we survey the recent results of epitaxial BiFeO_3_ ultrathin films to illustrate how the atomic structure and phase are markedly influenced by the interface between the film and the substrate, and to emphasize the roles of misfit strain and depolarization field on determining the domain patterns, phase transformation and associated physical properties of BiFeO_3_ ultrathin films, such as polarization, piezoelectricity, and magnetism. One of the obvious consequences of the misfit strain on BiFeO_3_ ultrathin films is the emergence of a sequence of phase transition from tetragonal to mixed tetragonal & rhombohedral, the rhombohedral, mixed rhombohedral & orthorhombic, and finally orthorhombic phases. Other striking features of this system are the stable domain patterns and the crossover of 71° and 109° domains with different electrical boundary conditions on the film surface, which can be controlled and manipulated through the depolarization field. The external field-sensitive enhancements of properties for BiFeO_3_ ultrathin films, including the polarization, magnetism and morphotropic phase boundary-relevant piezoelectric response, offer us deeper insights into the investigations of the emergent properties and phenomena of epitaxial ultrathin films under various mechanical/electrical constraints. Finally, we briefly summarize the recent progress and list open questions for future study on BiFeO_3_ ultrathin films.

## 1. Introduction

Nowadays, there is a growing demand for materials exhibiting various functional electronic properties for plenty of applications involving sensing, actuation and information/energy storage. A fundamental basis for understanding the occurrence of these intriguing functional properties in materials is the evolution of their symmetry as functions of external stimuli such as temperature, stress, electrical/magnetic field and pressure [1], which is subsequent to phase transitions due to the broken symmetry. In terms of the time reversal and the spatial inversion, materials can be mainly classified as ferromagnets, ferroelectrics, ferroelastics and ferrotoridics (*i.e.*, ferroic) [2]. Multiferroics are materials that simultaneously possess more than one (anti-) ferroic order parameter. Recently, multiferroic materials have attracted great attention in the development of advanced functional electronic devices [3,4,5,6], due to the coupling among various order parameters, particularly the magnetoelectric coupling between ferroelectric and magnetic order parameters.

The search for multiferroic materials has led to renewed interest in perovskites. Among them, lead-free BiFeO_3_ (BFO) [7], which exhibits both ferroelectric and antiferromagnetic at room temperature, is one of the most studied materials due to its advantages such as high Curie and Néel temperatures (*T*_c_ and *T*_N_), robust spontaneous polarization (*P*_s_ = ~100 μC/cm^2^ along <111> direction) and relative simple perovskite structure [8,9]. For bulk BFO material, it undergoes a phase transition from paraelectric to rhombohedral ferroelectric (*a*_pc_ = 3.965, α_pc_ = 89.35°) with the *T*_c_ (~1103 K) [10]. Meanwhile, BFO is a G-type antiferromagnet with *T*_N_~643 K. The simultaneous ferroelectric and anti-ferromagnetic orders in BFO currently are giving rise to enormous interest in the implementation of the coupling between the electrical and magnetic orders [4]. More recently, interest in BFO thin films has increased tremendously due to the interfacial effect between the film and the substrate. The interface induced new exotic behaviors of BFO thin films are of scientific interest and technological importance for applications and raises fundamental questions on understanding the roles of external mechanical and electrical constraints near the interface, on manipulating the atomic structure, phase transition and corresponding functional properties. In the present paper, we review the progress in this direction and propose possible areas of future research.

Compared with the bulk counterpart, it has been proven that there exist many new phenomena in epitaxial heterogeneous ferroic films [11]. For instance, the crystal structure, phase (or domain) and functional properties, such as magnetic, polarization and piezoelectric are changed dramatically, due to external effects such as the misfit strain resulting from the lattice mismatch between the constrained film and the underlying substrate. The appearance of various domain structures in ferroelectric thin films is ascribed to the surface energy on the film surfaces, *i.e.*, the depolarization field energy. By decreasing the depolarization field, the stable domain pattern could transform from a multi-domain pattern to a mono-domain one in tetragonal ferroelectric films or from a 109° domain to a 71° one in rhombohedral ferroelectric films, with different conduction and other transport properties.

Multiferroic BFO has been the subject of intense study ever since it was discovered, and several reviews on domain structures and related properties are available [4,7,12,13,14,15,16]. In the present review, particular emphasis is placed on investigations of epitaxial BFO ultrathin films, since new phase, novel ferroelectric, ferromagnetic and piezoelectric properties emerge while keeping the extrinsic factors such as electrical/mechanical boundary conditions at the interface between the film and the substrate under control. Specifically, we restrict ourselves to several fundamental aspects of strained BFO ultrathin films, in the light of recent developments. (1) The structure and corresponding phase transition are the most fundamental factors in ultrathin electronic films, which provide important insights into many critical phenomena and physical properties of BFO ultrathin films. (2) The relationship between structure-properties of BFO ultrathin films will be discussed. After discussing the extrinsic effect of misfit strain on crystal structure and corresponding phase transition of BFO films, we will illustrate how properties such as ferroelectricity, ferromagnetism and piezoelectricity are affected markedly by extrinsic misfit strain. (3) Finally, effects of extrinsic electrical/mechanical constraints on stability and manipulation of rhombohedral domains of BFO films will be reviewed. Domain pattern (including domain stability, its size and coexistence and crossover of domains) is another fundamental aspect of BFO films, which is closely connected to most macroscopic properties (polarization, piezoelectricity, conductivity, and photovoltaic *etc.*).

## 2. Effect of Misfit Strain on BFO Ultrathin Films

### 2.1. Misfit Strain in Epitaxial Ferroic Ultrathin Films

Recently, strain engineering has become a widely accepted and effective technique to tune and manipulate the behavior and properties of functional oxide thin films [17,18]. Misfit strain is mainly formed through differences of lattice parameters between the film and the underlying substrate, as well as the mismatch of thermal expansion coefficients between the film and the substrate. An obvious way to adjust misfit strain in the hetero-epitaxial structure is by selection of proper substrate materials.

Generally, suitable substrate materials are limited to ensure epitaxial growth due to the simultaneous considerations of reasonable match of their lattice constants and the thermal expansion coefficients between films and substrates. The film is usually strained and the total strain energy is sensitive to the film thickness. The total strain energy increases for a thicker film. For a sufficiently thin film, the misfit strain energy is lower than the energy cost for generating defects, such as misfit dislocation or domains, and the film is fully clamped by substrates. Above a certain critical thickness *h*_c_, it becomes more favorable to create misfit dislocations or other defects in films. When epitaxial thin films exceed the critical thickness *h*_c_, misfit dislocations are generally formed and the misfit strain can be partially or completely relaxed in the hetero-structures [19]. Meanwhile, the larger the lattice mismatch between the film and the substrate, the smaller the critical thickness *h*_c_ for strain relaxation. To comprehensively investigate the effect of misfit strain, particularly for high-strained films, the thickness of epitaxial BFO films as reviewed here has been restricted to a few tens of nanometers. With regard to BiFeO_3_/SrTiO_3_ systems with small misfit strain ~1.4%, the critical thickness is ~30 nm, below which the strain results in changes of structure and related properties of BFO films [20,21,22]. Regardless of the differences of the thermal expansion coefficient, misfit strain arises mainly due to lattice mismatch at the film/substrate interface when films are epitaxially grown on the substrate. There are two types of misfit strains at the film/substrate interface: Normal strain and shear strain. The normal strain is a deformation caused by normal forces such as tension or compression that acts perpendicular to the cross-sectional area, while the shear strain is a deformation obtained from forces acting tangential to the cross-sectional area. The magnitude of the in-plane normal misfit strain can be estimated based on the differences of the lattice parameters between the film and the substrate, which can be expressed as ε = (*a_s_*−*a*_0_)/*a_s_*, where *a*_s_ is the lattice parameter of the substrate and *a*_0_ is the lattice parameter of the film. Although it is inevitable in epitaxial films, the occurrence of the misfit strain has been proven to have significant impacts on the modifications of structure and functional properties of ferroic thin films, particularly in the phase transition and the associated dielectric/piezoelectric properties [17,23].

Due to the strong sensitivity to misfit strain, plenty of studies have investigated the influence of the biaxial strain on the temperature-misfit strain phase diagrams for ferroelectric films. Based on the phenomenological theory, the possible phases, polarization directions, and associated crystallographic symmetries of ferroelectrics are summarized and listed in Table 1 [24]. The c phase (*P*_1_ = *P*_2_ = 0, *P*_3_ ≠ 0) is a tetragonal (T) with a symmetry P4 mm, while the aa phase (*P*_1_ = *P*_2_ ≠ 0, *P*_3_ = 0) is an orthorhombic (O) C2 mm one. Notably, the symmetry of a phase (*P*_1_ ≠ 0, *P*_2_ = *P*_3_ = 0) is not tetragonal but orthorhombic. The r phase is a monoclinic (M) phase and has two sub-phases (M_A_ and M_B_), which can be identified by the magnitude of the in-plane and out-of-plane components of the polarization. For the M_A_ phase, where *P*_1_ = *P*_2_ <*P*_3_, it has a larger out-of-plane polarization *P*_3_, in comparison with that of M_B_. In analogy to M_A_ and M_B_, the M_C_ notation stands for the phase in space group Pm with polarization components along *P*_1_ ≠ 0, *P*_2_ = 0, *P*_3_ ≠ 0.

**Table 1:** Schematic representation, polarization components, notations, symmetry, space group and basis vectors in the mono-domain phase model. (Adapted with permission from [24], Copyright 2009 Springer)

	Polarization components	Notation	Symmetry	Space group	Basis vectors
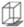	P_1_ = P_2_ = P_3_ = 0	paraelectric	Tetragonal	P4/mm	[100], [010], [001]
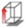	P_1_ = P_2_ = 0, P_3_ ≠ 0	c phase	Tetragonal	P4mm	[100], [010], [001]
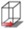	P_1_ ≠ 0, P_2_ = P_3_ = 0	a phase	Orthorhombic	P2mm	[100], [010], [001]
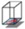	P_1_ = P_2_ ≠ 0, P_3_ = 0	aa phase	Orthorhombic	C2mm	[110], [110], [001]
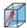	P_1_ = P_2_ ≠ 0 < P_3_ ≠ 0	r phase ( M_A_)	Monoclinic	Cm	[110], [110], [001]
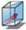	P_1_ = P_2_ ≠ 0 > P_3_ ≠ 0	r phase ( M_B_)	Monoclinic	Cm	[110], [110], [001]
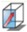	P_1_ ≠ 0, P_2_ = 0, P_3_ ≠ 0	ac phase ( M_C_)	Monoclinic	Pm	[100], [010], [001]
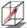	P_1_ = P_2_ = P_3_ ≠ 0	F_o_	Monoclinic	Cm	[110], [110], [001]

### 2.2. Normal Strain and Structural Phase Transition of BFO Ultrathin Films

**Figure 1:**
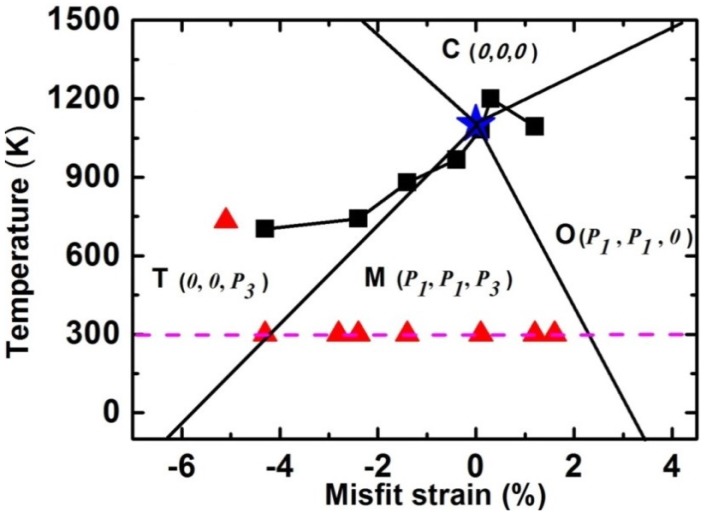
Misfit strain and temperature-dependent phase transition of strained BiFeO_3_ (BFO) films (Data adapted from [22,29,30,35,48,49], Star symbol denotes *T*_c_ of bulk BFO; Triangle symbols denote strain induced phases at room temperature; Square symbols denote *T*_c_ of strained BFO films).

In analogy to ferroic films [17,23], plenty of studies show that the misfit strain can be employed to tune various phases and functionalities such as ferroelectric, ferromagnetic and piezoelectric properties of multiferroic BFO ultrathin films [22,25,26,27,28,29,30,31,32,33,34,35,36,37,38,39,40,41]. Figure 1 shows the recent calculated and experimental strain-temperature phase diagram of BFO films [28,42,43]. There it is shown by plotting that there is a sequence of phase transition in the examined misfit strain range, from the tetragonal (T) phase, the monoclinic (M_C_, M_A_, and M_B_) phase to the orthorhombic (O) phase. As the misfit strain increases, the corresponding strain-dependent out-of-plane polarization *P*_3_ decreases [22,28,37,42,43], For compressive strains, early density functional theory predicts that a monoclinic Cc structure is energetically more favorable than the tetragonal P4mm one for BFO films grown on substrates with compressive strain larger than 4% [41,44], which has been experimentally confirmed [27,30,31,40]. Meanwhile, phenomenological results show that a large compressive normal strain (ε*_xx_* <−4.3%) favors the occurrence of T phase with only the non-zero vertical polarization component (*P*_1_ = *P*_2_ = 0, *P*_3_ ≠ 0) [21,22,28]. T phase starts to relax to R phase by reducing the magnitude of misfit strain or the dimension of strain constraints [22,29,30,31,45,46]. Meanwhile, both experimental and theoretical studies show that the relaxation is much easier in uniaxial constraint films than in biaxial ones [46]. Notably, there is a mixed T&R phase near the T/M boundary due to very close free energies for the intermediate compressive misfit strain, accounting for the observed abnormal dielectric and piezoelectric properties near the two phase boundaries [28]. Moreover, the equilibrium phase boundary such as the distortion of phases and the orientation of domain interfaces between T and M (R-like) are quantitatively analyzed and determined based on the “dense domain” model [28,47].

**Figure 2:**
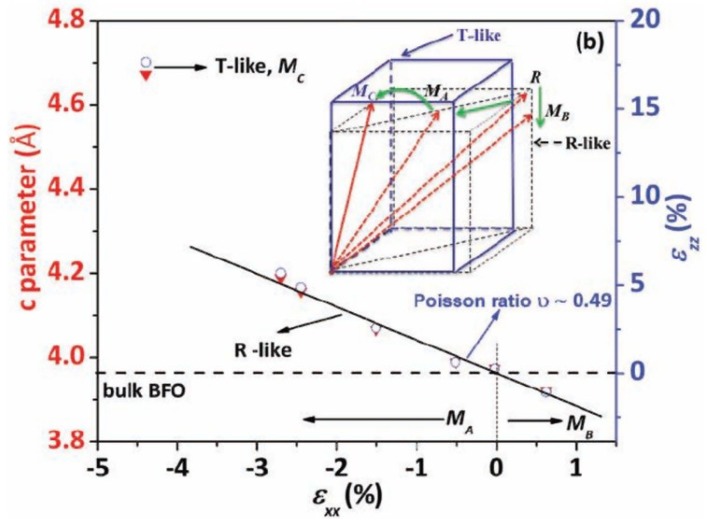
The out-of-plane lattice parameters (red) and ε*_zz_* lattice strain (blue) as a function of in-plane misfit strain ε*_xx_*. Inset is the possible strain-induced rotation path of polarization directions. (Reprinted with permission from [30]. Copyright 2011 WILEY-VCH)

Experimentally, compressive strain induced structure/phase transitions for BFO ultrathin films on various substrates (such as SrTiO_3_ (STO), NdGaO_3_ (NGO), LaAlO_3_ (LAO) and YAlO_3_ (YAO)) have been studied intensively [22,26,27,29,30,31,32,36,46,48,50,51,52,53]. Using piezoelectric force microscopy (PFM), transmission electron microscopy (TEM) and synchrotron X-ray reciprocal space maps (RSM), the strain-driven phase transitions (R → M_A_ → M_C_ → T) have been confirmed and are demonstrated in Figure 2. It is worth noting that there is a M_A_ → M_C_ phase transition for highly strained BFO and Sm-doping BFO ultrathin films when grown on LAO (with misfit strain ε*_xx_*~−4.3%) [27,30,54], rather than a simple iso-symmetric one (M_A_ → M_A_) [22,26,41]. For thicker films [51,55], the compressively strained film starts to relax and a mixed T&R phase is formed. Compared to the conventional substitution-induced morphotropic phase boundary (MPB) in solid solution system, the strain-driven mixed phase gives rise to a stable MPB with large dielectric and piezoelectric responses in BFO films [22,52]. Furthermore, more recently comprehensive synchrotron XRD studies have revealed that the mixed phase region of epitaxial BFO/LAO hetero-structures consists of two lower symmetry triclinic phases with slight energy gaps between the two triclinic phases [32], which could shed some light on the mechanism for the enhanced piezoelectric response near the strain-induced MPB.

**Figure 3:**
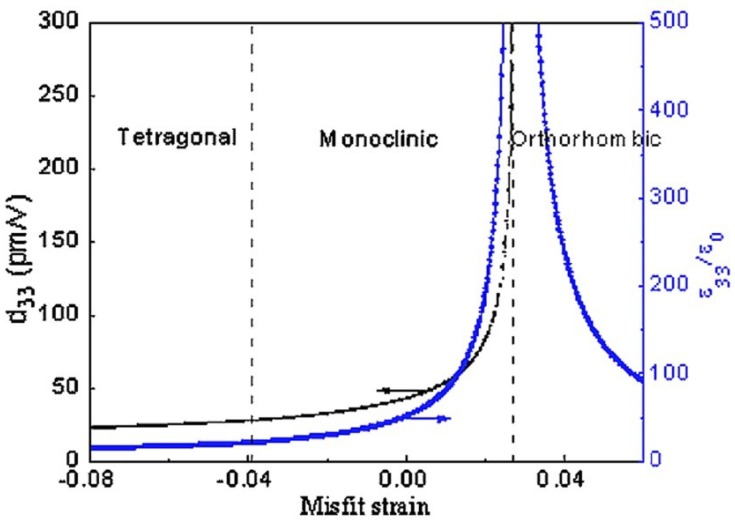
Misfit strain induced phase transition and associated dielectric/piezoelectric responses of BFO films. (Reprinted with permission from [28]. Copyright 2010 American Institute of Physics)

In parallel, there have been several attempts to investigate the effects of tensile strain on phase transition and related physical properties in BFO films. Pioneer theoretical works indicate that tensile strains can induce phase transitions for rhombohedral BFO films, with the transformation from R to M_B_, and finally to orthorhombic phase [22,28,33,34,35]. Based on first principles calculations [33], it has been reported that a large tensile strain (ε*_xx_*~8%) might induce an orthorhombic phase. In contrast, phenomenological calculations show that a relative small tensile strain range (ε*_xx_*~1.25%–2.7%) could result in the orthorhombic phase [21,22,28,34]. Furthermore, the phenomenological results indicate there is another tensile strain induced mixed rhombohedral and orthorhombic phase with abnormal giant dielectric and piezoelectric responses for BFO films deposited on a tensile substrate with ε*_xx_* = 2.7% [28]. The strain-induced dielectric response shown in Figure 3 exhibits a sharp anomaly, which corresponds to the excellent intrinsic piezoelectric response 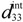 near the mixed phases. The value of 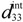 is much larger than that of the compressive strain-induced one (60 pm/V for rhombohedral phase and 120 pm/V for mixed phase) [22,52]. Thus, it implies that there is another tensile strain induced MPB-like behavior near the M and O phase boundaries. One example of the unconventional MPB occurring near the O phase has been reported in the (1 − *x*)NaNbO_3_-*x*CaTiO_3_ system [56], which lies between an O structure with the Pbma space group and the other O structure with the Pbnm space group. Experimentally, it has been demonstrated that BFO films deposited on small tensile strain substrates (such as GdScO_3_ and SmScO_3_) possess a M_B_-type monoclinic structure [29,35,57]. For larger tensile strain (NdScO_3_ with misfit strain ~1.5%), Yang *et al.* [34] claimed that an orthorhombic BFO could be achieved through a combination of controlling electrical boundary conditions of the films surface. However, recent experimental results from Chen *et al.* [35] reveal that, even deposited on the commercially available largest tensile PrScO_3_ (PSO) substrate, only a monoclinic M_B_ phase is exhibited, which indicates that a larger tensile strain is required to realize the predicted strain-induced orthorhombic phase (aa phase) for BFO films. More recently, a mixed rhombohedral and orthorhombic phase has been demonstrated experimentally, by means of a phase separation for epitaxial BFO films grown on PSO substrate [58]. Thus, there are still certain discrepancies between theoretical and experimental achievements regarding the critical tensile misfit strain on the occurrence of the orthorhombic phase of BFO films.

**Figure 4:**
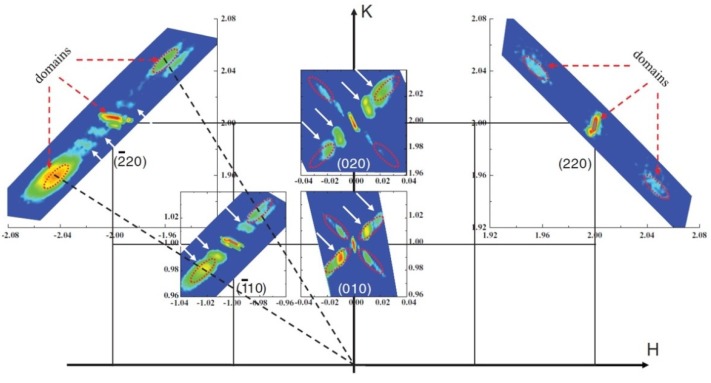
In-plane reciprocal space mappings (RSMs) of 10-nm thick BFO film grown on LaAlO_3_ (LAO). Red arrows and red circles denote the reflections of domain variants while white arrows denote periodic modulation. (Reprinted with permission from [36]. Copyright 2013 American Physical Society)

Besides the strain-induced phase transitions and mixed phases, various domain patterns and their periodic modulation have been revealed in strained BFO ultrathin films based on synchrotron grazing incidence X-ray diffraction (GIXRD) technique [36]. Figure 4 shows that the modulations due to periodic domains is along <110> directions in the strained-induced T-like phase of 10-nm thick BFO films grown on LAO, which are perpendicular to the domain walls directions. These findings widen the knowledge of phase transition and domain or crystal structure of strained BFO films and could shed lights on the mechanism of strain-induced enhanced piezoelectric response near MPB.

### 2.3. Shear Strain and Occurrence of M_C_ Phase of BFO Ultrathin Films

**Figure 5:**
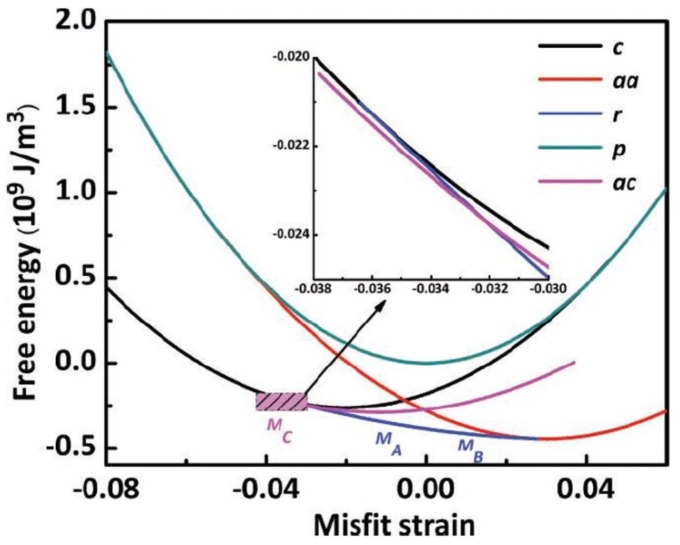
Effect of the shear strain from BFO film itself on the equilibrium of the phase transition. (Reprinted with permission from [30]. Copyright 2011 WILEY-VCH)

**Figure 6:**
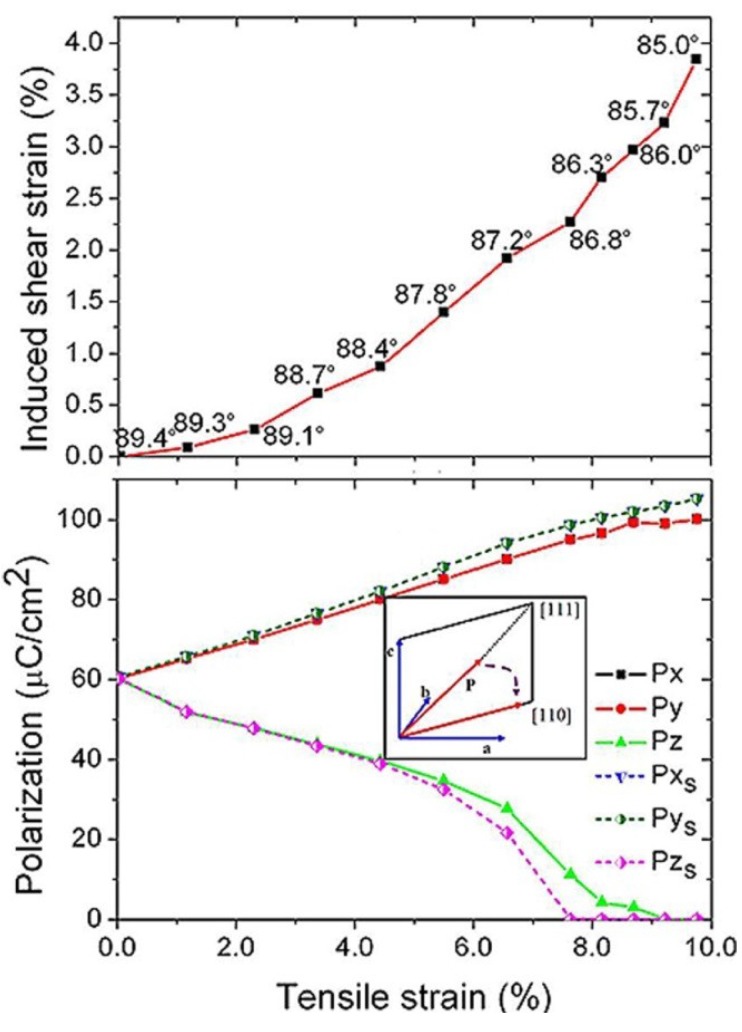
The tensile strain induced shear strain (the numbers in the Figure denote the in-plane angle corresponding to the most stable structure under tensile strain) and the polarization under tensile strain. (Reprinted with permission from [65]. Copyright 2014 Macmillan Publishers Limited)

We have reviewed the effect of the normal strain on the phase transition and associated physical properties of epitaxial BFO films. Now, we consider the shear strain effect of BFO films. Usually, the deposition temperature *T*_G_ of most ferroic films is higher than that of the Curie temperature *T*_c_. Thus, the films are generally at a cubic phase when deposited on the substrates. The lattice mismatch between the cubic film and the cubic substrate only leads to the occurrence of normal misfit strain for these epitaxial hetero-structures. However, a shear strain effect occurs when the cubic ferroic films is deposited on the rhombohedral substrates such as GaN. In comparison with the effect of normal misfit strain, it has been shown that the shear strain from the substrates could induce significant shifts of the stable points for phase transitions and the occurrences of extra novel properties in ferroelectric PbTiO_3_ films [59,60]. On the other hand, it is worth noting that there is another shear strain for certain rhombohedral materials, which is not from the substrate but from the films themselves. These materials have higher *T*_c_ from the cubic to the rhombohedral phase [61,62,63,64], compared to that of the deposition temperature *T*_d_. As a consequence, once it is deposited on the substrate, the material is already a rhombohedral phase and there is an angle mismatch with α_pc_ (α_pc_≠ 90°) between the rhombohedral film and the cubic substrate, which results in the in-plane shear strain for epitaxial rhombohedral films. For BFO (*T*_c_~1100 K and *T*_d_~1000 K), a rhombohedral phase with pseudocubic lattice parameters α_pc_ = 0.396 nm and α_pc_ = 89.4° is formed when the film is deposited on the substrate, which results in the occurrence of shear strain ε*_xy_* between the film and the cubic substrate. The magnitude of ε*_xy_* for BFO can be calculated as ε*_xy_* = π(α_pc_/90−1)/2 = −1.0%. It is seen from Figure 5 that the shear strain has obviously influenced the stable phase diagram of BFO films. Compared to the normal strain induced phase transition as described in the previous section, a new stable ac (*P*_1_ ≠ 0, *P*_2_ = 0, *P*_3_ ≠ 0) phase arises (as shown in the inset of Figure 5) between the T phase and M_A_ phase with the compressive normal strain about −4%, which agrees well with the experimental observation of monoclinic M_C_ phase for epitaxial BFO thin films deposited on LAO. Moreover, the recent first-principles calculations further predict the shear strain effect for the tensile strained BFO films (Figure 6) [65]. Remarkably, the shear strain increases under larger tensile substrates, which induces richer phases, and enhances related functional properties in BFO films

### 2.4. Misfit Strain Induced Enhancements of Polarization, Magnetic and Piezoelectric Responses of BFO Thin Films

**Figure 7:**
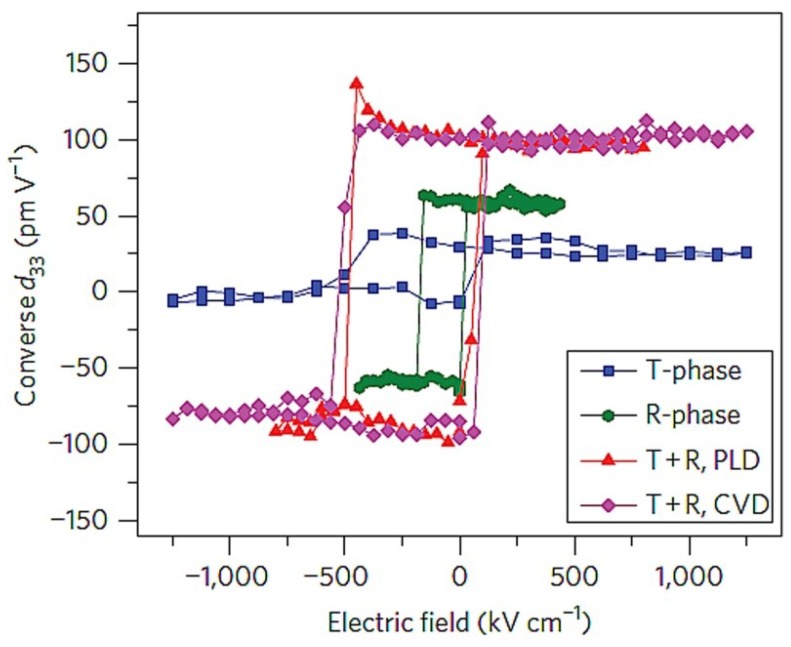
Piezoelectric hysteresis loops from T-like (blue curve), R-like (green curve) and mixed-phase (purple and red curves) BFO thin films. (Reprinted with permission from [52]. Copyright 2011 Macmillan Publishers Limited)

**Figure 8:**
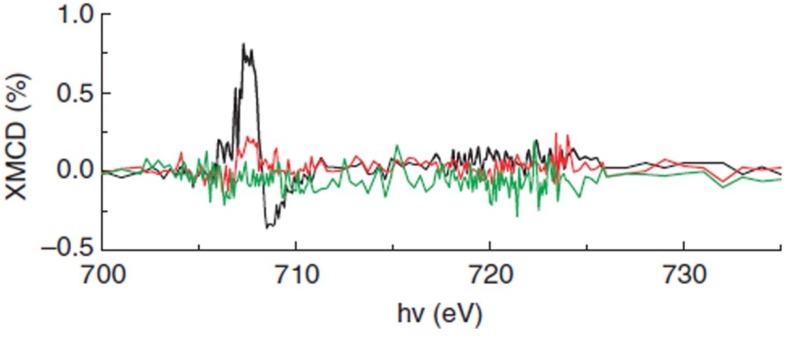
X-ray magnetic circular dichroism study of different phases of BFO thin films. (Reprinted with permission from [70]. Copyright 2011 Macmillan Publishers Limited) Mixed phase (black), pure rhombohedral phase (red) and pure tetragonal phase (green).

Accompanying strain-induced structure and phase transformations, numerous enhanced functional properties of BFO ultrathin films have been progressively revealed. When deposited on the compressive substrates [21,28,37,42,43], the total polarization of epitaxial BFO films can be substantially enhanced. With the increase of the compressive strain, the polarization is predicted to increase from 100 to 160 µC/cm^2^ with the phase transition from rhombohedral to tetragonal, which has been experimentally confirmed with the strain-induced enhancement of polarization up to ~130 µC/cm2 for highly compressively strained BFO films [52,66]. It has also been shown that the misfit strain can induce an unconventional strain-induced MPB with dielectric/piezoelectric enhancements near the MPB [22,28,52]. A mixed tetragonal & rhombohedral phase is stabilized for BFO films deposited on the compressive LAO substrate, which results in a new MPB due to the negligible energy barrier between the tetragonal phase and the rhombohedral phase. Analogous to the conventional composition-dependent MPB in PZT [67,68,69], this strain-induced MPB accounts for the enhancement of longitudinal piezoelectric response *d*_33_ [52]. The corresponding value of *d*_33_ in the mixed phase for BFO/LAO hetero-films shown in Figure 7 is 120 pm/V, which is much larger than these of pure rhombohedral phase (60 pm/V) or tetragonal phase (30 pm/V) of BFO. Furthermore, due to the strain-induced phase transition, the magnetic property of BFO films is subsequently changed. Contrary to G-type antiferromagnetic ordering in bulk part, there are several works elaborating the strain-driven magnetic response of tetragonal phase, mixed phase and fully relaxed rhombohedral phase of strained BFO films [70,71,72]. It has been revealed that the magnetic property at the mixed phase of BFO films (Figure 8) is strikingly different from the parent phases (tetragonal and rhombohedral) and the spontaneous magnetization at the strain-induced mixed phase is significantly enhanced.

### 2.5. Abnormal Poisson’s Ratio in BFO

As described above, the strain-driven deformation of structure, phase transition and associated enhancements of BFO ultrathin films are highly sensitive to the magnitude of misfit strain at the interface between the film and the underlying substrate. For example, a larger compressive misfit strain leads to a corresponding contraction of in-plane lattice and a greater elongation of out-of-plane lattice of epitaxial BFO films due to the positive Poisson effect. The Poisson’s ratio is defined as *ν* = ε_zz_/ε*_xx_*, where ε*_zz_* and ε*_xx_* stand for the deformation of out-of-plane and in-plane films, respectively, which offers a fundamental metric to compare the deformations of ε*_zz_* and ε*_xx_*. Poisson’s ratio *ν* is thought to be a constant and the value is typically in the range of 0.2–0.4 for perovskites (for BFO, *ν~*0.3 [73] For a given *ν*, the magnitude of out-of-plane deformation ε*_zz_* of epitaxial films can be tuned by the in-plane misfit strain ε*_xx_*, which is determined by selecting different substrates. For epitaxial BFO films, the in-plane misfit strain ε*_xx_* can change from compressive 6.8% to tensile 1.5% by choosing proper substrates (such as YAlO_3_, PrScO_3_), which results in a sequence of structure deformations, phase transitions and occurrences of new properties of BFO films. In comparison with the thought positive constant, recent results reveal that the Poisson’s ratio (PR) in perovskites is an anisotropic variable [74]. This demonstrates that the PR of perovskites is highly sensitive to the crystallographic axes of materials and the value of it could become negative, zero or positive along certain directions. Furthermore, an unexpected large Poisson’s ratio (~0.49) of BFO films has been reported [30], in sharp contrast to the previous values for polycrystalline BFO (~0.3) or other perovskites (~0.2–0.4) [73]. These findings might offer insights into understanding and manipulation of mechanical-related multifunctional properties, including the strain-induced polarization, magnetism and piezoelectric response in epitaxial functional oxide films.

## 3. Temperature-Dependent Phase Transitions of Epitaxial BFO Thin Films

**Figure 9:**
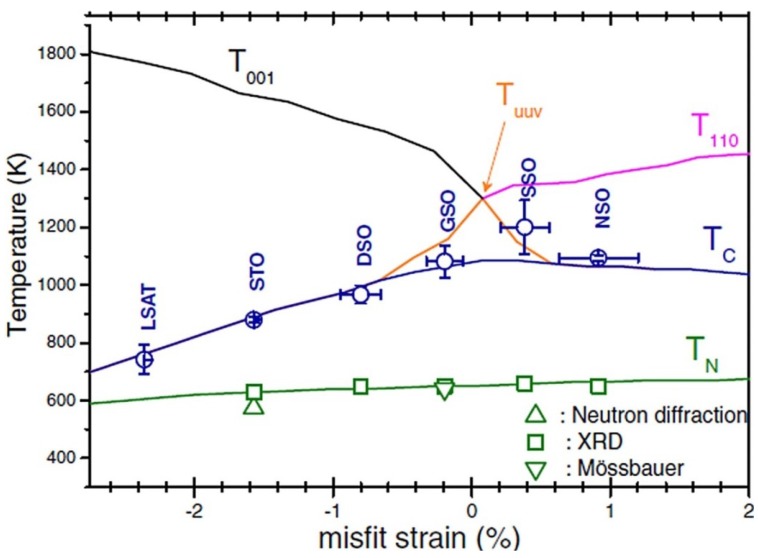
Transition temperature of BFO film as a function of misfit strain: theoretical *T*_c_ (blue line) and *T*_N_ (green line); experimental *T*_c_ (circles) and *T*_N_ (squares and triangles) values; the activation temperatures for the antiferrodistortive oxygen tilting along *z* direction (tetragonal distortion *T*_001_, black line), *x*-*y* plane (orthorhombic one *T*_110_, red line), and for the tilting along [uuv] direction (monoclinic distortion *T*_uuv_, orange line) are plotted. (Reprinted with permission from [29]. Copyright 2010 American Physical Society)

**Figure 10:**
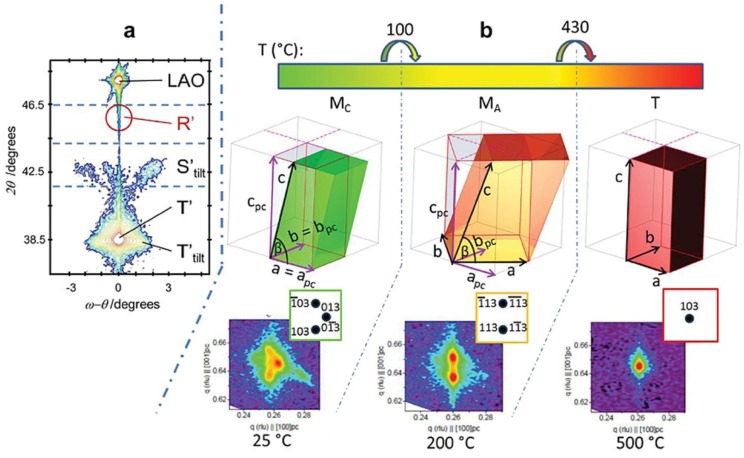
(**a**) X-ray diffraction map for a 45-nm thick BFO film on LAO, the peaks of the LAO substrate, and the polymorphs of film (S’_tilt_, T’ and T’_tilt_) are labeled; (**b**) temperature dependence of the structure of the T’ polymorph. (Reprinted with permission from [49]. Copyright 2013 WILEY-VCH)

It is notable that temperature also plays a key role in the phase transition of multiferroic BFO thin films. BFO experiences a phase transition from a paraelectric to a rhombohedral ferroelectric phase on cooling. There are already many studies on the temperature-dependent phase transitions in BFO bulk counterpart [7,62,63]. For epitaxial BFO thin films, recent reports disclose richer temperature-induced phase transitions from cubic paraelectric to various ferroelectric phases such as tetragonal, M_A_ or M_C_ phases [29,48,49]. Meanwhile, the Curie temperature *T*_c_ of BFO films also varies with the misfit strain. Theoretically, it was predicted that *T*_c_ of BFO from paraelectric to ferroelectric increases with larger misfit strain [21,22], which is similar to the previous results in BaTiO_3_ films [75]. However, the recent in-depth experimental studies obviously reveal that *T*_c_ of strained BFO films unexpectedly decreases for larger compressive misfit strains shown in Figure 9 [29,31,36], which may be attributed to an interplay of polar and oxygen tilting instabilities [29,49]. Furthermore, more recent experimental efforts show a richer temperature-induced phase transition diagram in BFO thin films. As temperature increases (Figure 10), phase transitions from M_C_ → M_A_→ T → C in BFO/LAO or BFO/LaSrAlO_4_ epitaxial systems are experienced, rather than the simple T → C as previously thought [36,48,49]. These results could open up ways to better understand and manipulate the temperature-dependent phase transition and related functional enhancement for epitaxial ferroic thin films.

## 4. Dependence of Depolarization Field and Stability of Domains in BFO Thin Films

The existence of spontaneous polarization is one of the main characteristics of ferroelectric materials [76], which are accompanied by the existence of surface energy (*i.e.*, the depolarization field energy) on the film surfaces. The depolarization field usually occurs due to the unscreened charges on the film surfaces or the inhomogeneous polarization distribution of the ferroelectric films, which is anti-parallel to the spontaneous polarization direction. It is energetically difficult for films to sustain a uniform polarization and a multi-domain structure is formed to reduce the depolarization field energy in ferroelectric films. It has been shown that the properties such as *T*_c_, the stability of domains and the crossover of multi/mono-domain patterns are, to a large extent, determined by the depolarization filed for ferroelectric films [77,78,79,80,81,82].

### 4.1. Stability and Crossover of Domain Patterns of BFO Thin Films

**Figure 11:**
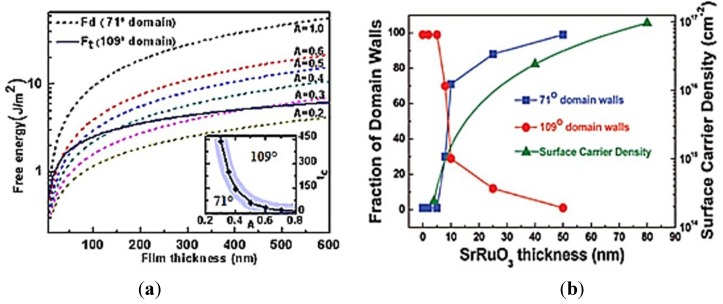
Effect of depolarization field on stability of 71° and 109° domains in BFO thin films. (**a**) Theoretical prediction of critical thickness *t*_c_ for the crossover from 109° to 71°; (**b**) Experimental evolutions of domain of La-BiFeO_3_ thin films as a function of SrRuO_3_ thickness (Reprinted with permission from [89,90]. Copyright 2011 American Institute of Physics; Copyright 2009 American Chemical Socienty)

Recently, intensive interest has been directed towards understanding the role of the depolarization field on rhombohedral BFO domain patterns and related properties such as electrical conduction, photovoltaic effect and magnetoelectric coupling [83,84,85,86,87,88]. To explore the multifunctional properties exhibited by the different nano-scale domains of BFO films, it is imperative to investigate the stability of rhombohedral domain patterns by controlling the closely correlated depolarization field. In order to quantize and manipulate the residual depolarization field energy, a simple but effective asymmetrical screen factor *A* is employed to describe the relationship between depolarization and stable domain patterns [89,90,91]. In combination with the film thickness, the effective screening coefficient *A* is used to manipulate the stable patterns between 71° and 109° domains for rhombohedral BFO thin films [90]. Figure 11 (left) shows a plot of the film thickness-depolarization field dependent domain diagram of BFO thin films. Due to the same direction of out-of-polarization, the depolarization field energy *F*_d_ of 71° domain pattern is much larger than the total energy of 109° domain pattern. Hence, the 109° domain pattern is more stable for open-circuited BFO thin films. On the contrary, it is generally a 71° domain pattern for short-circuited BFO films. As *A* decreases, more polarization charges near the film surface are compensated by free charges and consequently the depolarization field energy *F*_d_ decreases. For example, *F*_d_ decreases from 14.11 J/m^2^ for *A* = 1.0 to 3.13 J/m^2^ for *A* = 0.4. There is a critical value for *A* (approximately *A* = 0.4 for a 150 nm BFO thin film), at which the total energy of the 109° domain patterns equals that of the 71° ones. As *A* decreases further, a crossover from the 109° domain to the 71° one occurs, which is consistent with the experimental observations [89]. Meanwhile, the stable rhombohedral domain patterns are sensitive to the film thickness. With the reduction of film thickness to a critical thickness *t*_c_, the stability changes gradually from a 109° domain to a 71° domain. Near the critical thickness *t*_c_, there could be a range of thicknesses over which both the 71° and 109° domains may coexist (as shown by the shaded region inset in Figure 11).

There are several ways to circumvent the depolarization field near the film surface. One practical approach is to deposit the films on electrodes. For thicker electrodes, the surface carrier density increases, which could suppresses the depolarization field dramatically near the BFO film surface and result in a transformation of domain pattern from 109° domain to 71° domain as shown in the right of Figure 11.

**Figure 12:**
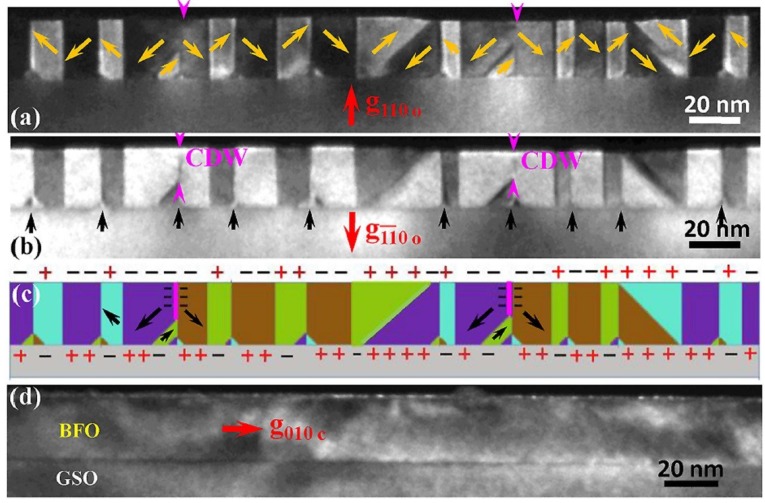
Dark-field transmission electron microscopy (TEM) images of a 24-nm thick BFO film on GSO substrate taken with near two beam (**a**) **g** = 110_0_ and (**b**) **g** = 110_0_; (**c**) a schematic of domain structures in (**a**) and (**b**); (**d**) dark-field image near [001]_o_ zone axis using **g** = 010_c_. (Reprinted with permission from [57]. Copyright 2013 American Institute of Physics)

Alternatively, it has been demonstrated that the depolarization field energy of BFO films at small strained BiFeO_3_/TdScO_3_ and BiFeO_3_/GdScO_3_ systems can be dramatically reduced by a self-assembly formation of vortex domains, rather than the deposition of electrodes, which has been clearly revealed recently by TEM near the BFO film surface (Figure 12) [53,57]. It should also be pointed out that different domain patterns, for instance, fractals or other irregular patterns rather than stripes [92], may be formed due to the different electric and mechanical boundaries, concomitantly, which result in a wide distribution of domain size and the divergence of the classical 1/2 power law relationship of domain size scaling behavior [57,92,93].

### 4.2. Domain Size Scaling Behavior for BFO Thin Films

**Figure 13:**
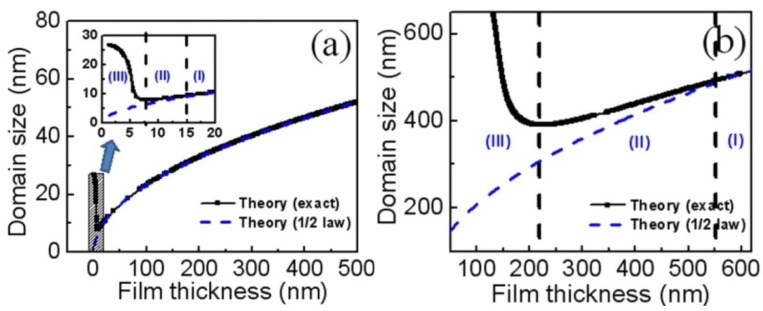
Thickness-dependent domain size scaling in 109° rhombohedral BFO films as (**a**) ferroelectric domains and (**b**) ferroelastic ones. (Reprinted with permission from [98]. Copyright 2013 American Institute of Physics)

The domain structure and its size in any ferroic, which also depend on various electrical and mechanical boundary conditions, are vital to the applications in data storage, actuator and sensors. The domain structure in rhombohedral BFO films could be rather complicated due to the possible eight polarization variants [12]. By introducing vicinal or anisotropic substrates, the polarization variants could obviously be reduced, which leads to the formation of 71° and 109° stripe domains [50,94,95]. Choosing appropriate electrical and mechanical boundary conditions, there are typically stripe 71° and 109° dense domain patterns for heterogeneous (001) BFO thin films [89,96]. For thick BFO films, the domain size decreases as the film thickness decreases, following classical square root behavior [97,98]. However, the thickness-dependent domain size scaling behavior starts to deviate from the conventional square root relationship for films less than a definite critical thickness (typically, below tens of nanometers), which leads to inconsistencies on domain size scaling behavior for BFO thin films with different thickness ranges [51,92,93]. Recently, Huang *et al.* [98] comprehensively investigated the thickness-dependent evolution of domain size in BFO thin films. The results show (Figure 13) that there are three regions for the domain size scaling behavior of BFO films: (I) a classical Landau-Kittle 1/2 law for thicker films; (II) the deviation from mean-field 1/2 law for intermediate film thickness; and (III) an increase exponentially in ultrathin films when decreasing film-thickness [98].

### 4.3. Domain Wall Properties of BFO Thin Films

In contrast to the domain, the domain wall itself also has unique structures and properties, such as phase transition in the domain wall [85], profile and size of the domain wall, conduction of the domain wall [84,86,99], ferroic or multiferroic [70] and photovoltaic of the domain wall [83], which could play crucial roles in future electronic devices. As one of the main characteristics and key components, the structure and properties of domain walls mainly influence and determine the overall properties of multiferroic BFO [38,85,100,101,102]. Meanwhile, it has been explicitly pointed out that there are plenty of emergent phenomena for domain walls in ferroic or multiferroic films. The changes in structure (particularly the electronic structure) at the domain walls can consequently lead to remarkable changes of physical properties: (1) conduction; (2) the interaction of ferroelectric and antiferromagnetic walls; and (3) photovoltaic responses of BFO ultrathin films [70,83,84,85,86,99,103,104]. Thus, it is believed that the understanding and manipulation of the domain wall patterns and the novel properties of the domain wall can stir up many new specific applications in ferroic/multiferroic materials.

## 5. Conclusions and Outlook

Despite the numerous works mentioned, in this review, we summarize here the effects of interfaces (including misfit strain and depolarization field) that significantly influence the domain structure, phase diagram and related properties of BFO ultrathin films. On the one hand, the misfit strain can be readily applied to epitaxial BFO films with the selection of appropriate substrates. This results in huge changes in structure and related properties of BFO films. For example, the normal strain induced enhanced polarization and magnetism; the strain-driven mixtures of tetragonal/orthorhombic and rhombohedral phases and enhanced piezoelectric responses. Furthermore, due to the higher *T*_c_ of BFO, the effect of shear strain from BFO itself has been highlighted, which has been observed experimentally, referring to the occurrence of M_c_ phase in the epitaxial BFO/LAO system. On the other hand, the depolarization field can have a tremendous effect on the stability and crossover of domains in BFO ultrathin films, and further manipulate the domain or the domain wall-related properties.

Although the studies of such systems have advanced considerably our understanding of the effects of the interface on giving rise to enhanced properties in BFO ultrathin films, we are still at the beginning of fully understanding these effects in epitaxial BFO films. Firstly, despite the profusion of interesting predicting strain-induced phenomena for BFO ultrathin films, the corresponding experimental achievements need to progress in parallel. For example, although theoretical studies have predicted that large tensile strain could induce an orthorhombic phase and another MPB-like behavior between rhombohedral and orthorhombic phases in BFO ultrathin films, so far there are few reports experimentally realizing it, mainly due to the lack of commercially availability of such large tensile substrates. Secondly, there are already intensive studies clearly addressing the strain induced phase transition and the extraordinary properties of BFO ultrathin films. However, the films are usually relaxed along the growth direction and strain gradients are inevitable between the film and the substrate or among different phases, in particular, for the highly strained BFO thin films with mixed phases. Thus, the strain gradient and the related intriguing phenomena, *i.e.*, flexoelectric and flexomagnetic effects, deserve further exploration in BFO thin films. Furthermore, in sharp contrast to the thought positive constant (~0.2–0.4) in ABO3 perovskites, the predicted tunable Poisson’s ratio with the range from negative to positive could offer new directions for strain engineering for epitaxial perovskite films, and particularly for BFO films on tensile substrates. Finally, advanced characterization tools are crucial to do further *in-situ* studies on temperature, electrical field and stress induced structure, phase transitions and physical properties of BFO ultrathin films.
